# Some Interesting Observations on the Doodle Bug

**Published:** 1911-10

**Authors:** 


Some Interesting Observations on the Doodle Bug.—Doctor, do you know what a doodle bug is? I dare say that every child in the South knows, and that when you were a child you put your mouth down close to the little doodle holes, and repeated, "doodle bug, doodle bug, doodle bug; your house is a-fire"; now, didn't you? But science does not know the doodle bug,—by that name, at least. In science, he is the larva of the ant-lion (*Myrmeleon obsoletus*), a *neuropterous* insect. The larva makes in the sand a pitfall to capture ants. He is a most insignificant beast, notwithstanding his aristocratic and high-sounding name and his apparent important place in natural history. He is a gray-back bug about the size of a split pea, and resembles an exaggerated louse. He is an old bachelor, a solitary bug,—lives alone and seemingly for the sole purpose of catching and eating ants. Evidently, he has a powerful appetite, for the ant is as big as he is, and it looks to me that if the ant had any sand in his gizzard he could whip,—even kill,—Mr. Doodle with one crunch of his formidable maxillary pinchers. But the ant gets rattled,—demoralized,—and his sole aim seems to be to escape,—to get out of the hole he has gotten into. Are there any human doodle bugs that get into holes and struggle against fate?

Mr. Doodle Bug lives at the bottom of his little dust-hole, *cache*—(he never shows himself)—and woe to the luckless ant that falls into it. As he struggles to climb out, Mr. Doodle rakes away the sand (dust) under his feet and he falls back again and again, when he is seized by the hind leg and, despite his strength and frantic struggles, he is pulled under. He is finally overcome, and Mr. Doodle then places his napkin under his chin and calmly sits down to a square meal. It is said that he only sucks the blood; but, during my recent camp experience of two months, when I watched the doodles for hours, I never saw a carcass thrown out. So, I suppose, he devours his prey—bones and all. It is as if a man should seize an ox by the hind leg, overpower him and eat him whole.

The amazing thing about the doodle bug is his prodigious strength! I have seen him hurl from his den fragments, say, of pecan hull, several times his size and ten times his weight. Relatively his strength is double that of Old Polyphemus, the Cyclops, and the missiles he handles with such ease are larger and heavier than the boulders Old Poly hurled at Ulysses and his crew, that time, you remember, when they literally *got into a hole* (the Cyclops' cave) in Sicily. I saw a doodle who had a fine dwelling-hole—scrupulously clean and well kept—hurl from his front porch a straw four inches long and an eighth thick. It would be about as if a man should throw a telephone pole across the street or over the house.

Now, this obscure gray-back bug hatches out,—somehow, sometime,—somewhere,—into a beautiful winged-fly—with *hymenopterous* (or, as the books say, *neuropterous*) wings. But it seems the bug is the lion, because the pretty, harmless fly belongs to the *ephemera*,—and only lives from one to three days! But in that time he, she or it, lives, moves and has its being—and gets in its work for another generation of doodle bugs;—a day,—which to them is relatively as long as our three score years and ten! How wonderful! How mysterious! How like human beings—in one respect! Do we not have male human beings who, lying *cache*, wait, watch for, capture and metaphorically devour the unwary? These "financiers" have been called "harpies," "birds of prey" and all sorts of hard names; but I suggest, as a more fitting and appropriate name, "human doodle bugs."

